# Serum vascular endothelial growth factor associated with the progression of granulosa cell tumor: a report of two cases

**DOI:** 10.1186/s13048-023-01197-z

**Published:** 2023-06-14

**Authors:** Kazuki Takasaki, Takayuki Ichinose, Yuko Miyagawa, Shiho Fukui, Kei Hashimoto, Haruka Nishida, Yuko Takahashi, Haruko Hiraike, Koji Saito, Yuko Sasajima, Kazunori Nagasaka

**Affiliations:** 1 Department of Obstetrics and Gynecology, Teikyo University School of Medicine, Kaga 2-11-1, Itabashi district, Tokyo, Japan; 2 Department of Pathology, Teikyo University School of Medicine, Tokyo, Japan

**Keywords:** Granulosa cell tumor, Vascular endothelial growth factor, Chemotherapy

## Abstract

**Background:**

Granulosa cell tumors (GCTs) account for approximately 2% of ovarian malignancies and are considered a rare type of ovarian cancer. GCTs are characterized by irregular genital bleeding after menopause due to female hormone production as well as late recurrence around 5–10 years after initial treatment. In this study, we investigated two cases of GCTs to find a biomarker that can be used to evaluate the treatment and predict recurrence.

**Case presentation:**

Case 1 was a 56-year-old woman who presented to our hospital with abdominal pain and distention. An abdominal tumor was found, and GCTs were diagnosed. Serum vascular endothelial growth factor (VEGF) levels decreased after surgery. Case 2 involved a 51-year-old woman with refractory GCTs. Carboplatin–paclitaxel combination therapy and bevacizumab were administered after the tumor resection. After chemotherapy, a decline in VEGF levels was observed, but serum VEGF levels increased again with disease progression.

**Conclusions:**

VEGF expression may be of clinical importance in GCTs as a clinical biomarker for disease progression, which may be used to determine the efficacy of bevacizumab against GCTs.

## Background

Granulosa cell tumors (GCTs) are rare tumors, representing 2–5% of all ovarian cancers [1]. Due to their rarity, detailed information on their pathogenesis and molecular characteristics is limited [2–4]. The standard treatment comprises debulking surgery, including hysterectomy and bilateral adnexectomy, for all patients with GCTs [1, 3]. Postoperative adjuvant chemotherapy is recommended in patients with advanced stage or stage I disease with high-risk factors, including tumor rupture, advanced age, menopause, and mitotic rate [3, 5]. Recently, a notable survey report on GCTs by Ebina et al. [6] reported the possibility of omitting diagnostic lymph node dissection for patients with pT1 during the initial surgery for pT1 GCTs. For patients with pT2 or higher, systematic dissection is required for the diagnosis of lymph node metastases. Careful observation of the abdominal cavity at the start of surgery is necessary to determine whether total dissection is required, especially in advanced cases with intraperitoneal dissemination, suggesting that achieving zero residual tumors or adequate tumor reduction is associated with improved prognosis. Thus, no residual tumors should be required in the surgical treatment of GCTs to avoid the recurrence. Nevertheless, evidence-based management of aggressive GCTs is limited, and GCTs tend to eventually recur after initial treatment [3]. Magnetic resonance imaging (MRI) is also used for diagnosis, but it has known limitations [7]. Angiogenesis reportedly plays a critical role in the development and progression of GCTs [2, 3], and overexpression of vascular endothelial growth factor (VEGF) has been reported to correlate with shorter disease-specific survival and poorer outcomes [3, 8], which may support the clinical benefits of anti-VEGF therapy [2, 8, 9]. Here, we present two cases of aggressive GCTs in which serum VEGF levels were measured and examined whether serum VEGF levels are predictive of recurrence.

## Case presentation

### Case 1

**Fig. 1:**
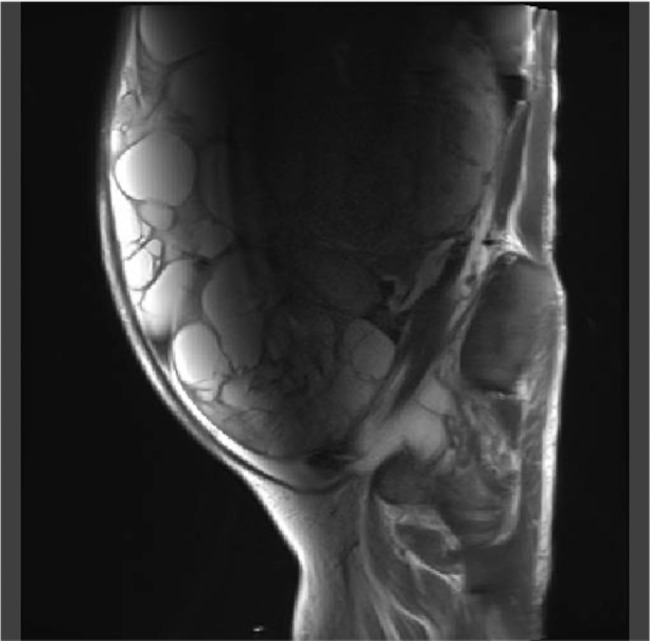
Magnetic resonance imaging (MRI) revealed an abdominal mass measuring up to 30 cm

**Fig. 2:**
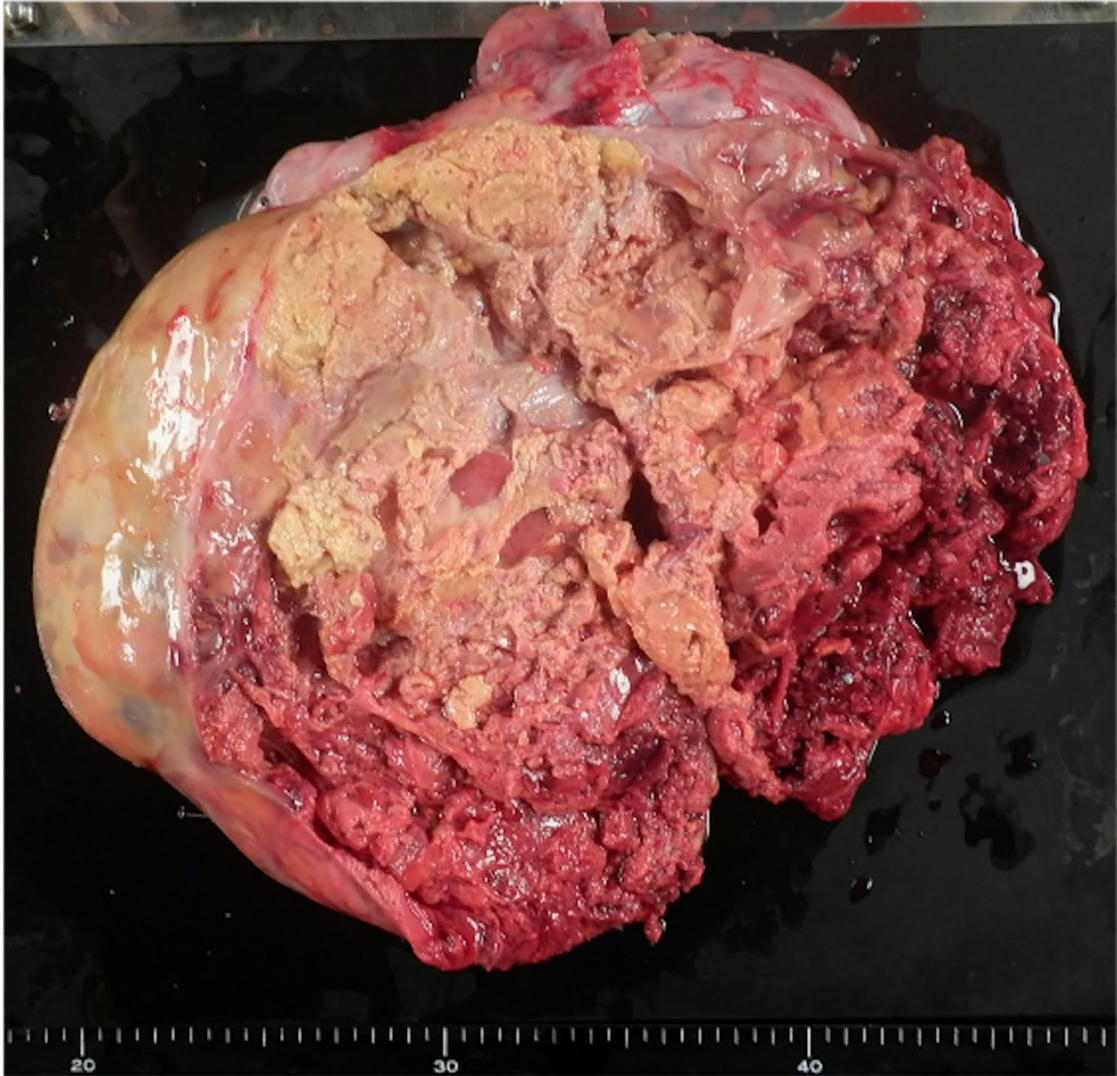
Gross appearance of the left ovarian tumor. The tumor had a diameter of 31 cm, with a white solid portion, hemorrhage, and necrosis

**Fig. 3:**
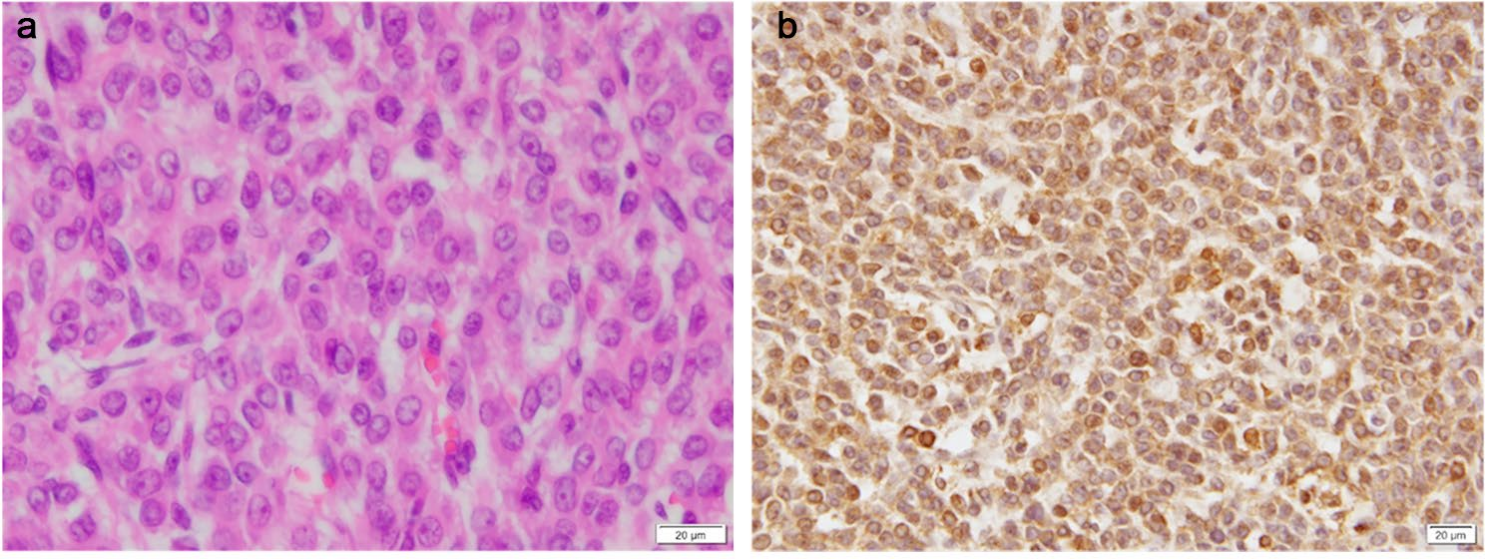
Microscopy findings of the tumor. (A) Diffuse proliferation of atypical cells with high N/C ratios, some of which have “coffee bean” nuclei (hematoxylin-eosin staining, *40). (B) Immunohistochemically, VEGF was diffusely and strongly positive in the tumor

A 56-year-old female presented to our hospital with severe abdominal distention, fatigue, nausea, and abdominal pain. She was initially taken to the emergency room. A chest x-ray showed significant pleural effusion in the right lung field, and an abdominal x-ray showed intestinal gas accumulation. She was admitted to the hospital for a thorough examination. She had no medical history or history of allergies. Blood tests performed on admission showed that the patient was severely anemic, with Hb of 5.6; therefore, six red blood cell transfusion units were administered and pleurodesis was performed to remove 1,500 mL of fluid. Cytology of the uterine cervix and endometrium was clear, and transvaginal ultrasonography revealed a substantial pelvic mass. MRI revealed an abdominal mass with a diameter of 30 cm (Fig. 1), ascites, and pleural effusion. Tumor marker levels were as follows: serum cancer antigen 125 (CA125), 776.1 U/mL; CA19-9, 17.9 U/mL; CEA, 2.0 ng/mL; and estradiol, 498.7 pg/mL. Computed tomography (CT) revealed no metastases. Enzyme-linked immunosorbent assay (ELISA) showed that the preoperative serum VEGF level was 1830 pg/mL. We performed total abdominal hysterectomy with bilateral salpingo-oophorectomy and omentectomy. We found approximately 4,000 mL of yellowish ascites and a left ovarian tumor adhered to the omentum and peritoneum. No abnormal findings were observed in other organs, including the uterus, right ovary, bilateral tubes, and omentum. In addition, no residual disease was observed in the abdomen after the surgery. Grossly, the left ovarian tumor measured 31 cm in diameter and contained a white solid portion with hemorrhage and necrosis (Fig. 2). Pathological findings revealed diffuse proliferation of atypical cells with high N/C ratios, some of which had “coffee bean” nuclei (Fig. 3A), which was pathologically consistent with GCTs. Immunohistochemically, VEGF was highly expressed in the tumor (Fig. 3B). One day after surgery, serum VEGF levels decreased to 750 pg/mL, as determined by ELISA. She was discharged eight days after surgery, and there has been no evidence of recurrence over the 4-year follow-up.

### Case 2

The patient was a 51-year-old female. She had undergone laparoscopic right salpingo-oophorectomy in another hospital 19 years prior and had been diagnosed with GCTs. She had undergone tumor resection at our institute for recurrence three times, and subsequently underwent total abdominal hysterectomy with left salpingo-oophorectomy and dissemination resection. Five years after the last surgery, recurrence was noted and tumor resection was performed again. After surgery, carboplatin-paclitaxel combination therapy (CP therapy) was administered. Before the administration of CP therapy, the serum VEGF level was 1680 pg/mL. Increased pleural effusion and ascites were found after the first cycle of CP therapy; therefore, bevacizumab, a monoclonal antibody against VEGF, was administered in combination with CP therapy. A reduction in ascites and pleural effusion was observed, and CP therapy with bevacizumab was continued. Two months after TC therapy, the serum VEGF level was 107 pg/mL. However, after three cycles of CP therapy, the serum VEGF level was slightly elevated (130.8 pg/mL), and CT revealed tumor dissemination and enlarged lymph nodes in the pelvis. Recurrence was suspected, but as the patient refused additional chemotherapy, palliative therapy was administered. The patient was alive at the 1-year follow-up after administration of CP therapy.

## Discussion and conclusions

Surgery is the standard initial treatment for GCTs, and complete tumor resection is recommended for all patients [4]. However, postoperative adjuvant chemotherapy has limited efficacy and novel treatments for GCTs are required. Indeed, although it has been reported that postoperative adjuvant chemotherapy is unnecessary in FIGO stage IC GCTs [10], Case 2 in this report showed repeated recurrences. It is well known that high VEGF expression is associated with survival of ovarian cancer [11]. In addition, previous studies have reported overexpression of VEGF in GCTs, both primary and recurrent types [9, 12]; thus, it has been associated with tumor angiogenesis and progression of GCTs. We have experienced two cases of GCTs with serum VEGF measurements, and we identified other cases of GTC diagnosed and treated in our institute, from the medical records. We summarized the VEGF immunohistochemistry results for GCTs in Table 1. A VEGF antibody (A-20, Santa Cruz Biotechnology, Tokyo, Japan) was used in the immunohistochemistry staining. The staining patterns of VEGF were almost homogeneous, and most cases were positive for VEGF in over 90% of tumor cells. The cases were divided into three groups with staining intensities of “moderate,” “high,” or “very high,” which was determined by a consensus between two researchers (K.S. and Y.S.). Both cases were positive for VEGF in tumor cells, and there was a tendency for strong staining with large tumors. Moreover, previous studies have reported that serum VEGF levels are elevated in cases of GCTs [9], and that there is no correlation between serum VEGF levels and tumor size or stage. In our study, serum VEGF levels were analyzed in two cases, and serum VEGF levels were almost high. These findings have been noted in previous studies [13, 14], but whether they predict recurrence is not well known. The limitation of this study is that it is a case report. However, the quantitative evaluation of serum VEGF levels over time in patients with advanced granulosa cell tumors, along with simultaneously conducted immunohistological studies may provide a new perspective on future treatment strategies for granulosa cell tumors.

**Table 1:** The immunohistochemistry of VEGF in 10 cases of granulosa cell tumors

Case number	Age (years)	Recurrence	Tumor size (cm)	Immunohistochemistry of VEGF	
				Staining area (%)	Staining intensities
1	48		5	90	high
2	43		6	100	moderate
3	40		6	100	high
4 (Case 2)	41	+	7	100	moderate
5	46		10	90	high
6	90		14	80	moderate
7	43		14	100	very high
8	53		14	100	very high
9	13	+	20	90	high
10 (Case 1)	56		24	100	very high

Abbreviations: VEGF, vascular endothelial growth factor.

Serum VEGF levels were higher in the primary tumor case than in the recurrent case in our analysis, which was consistent with a previous study [7], possibly due to an increased VEGF-producing tumor burden in the primary tumor case compared with that in the recurrent case.

As shown in our cases, serum VEGF levels decreased after treatment, which comprised surgery and chemotherapy, reflecting tumor cytoreduction and a decrease in ascites. A previous study reported a decrease in serum VEGF levels after tumor removal [15]; however, our case showed decreased serum VEGF levels after adjuvant chemotherapy, and elevated VEGF levels before detecting disease progression, which supports the clinical efficacy of VEGF in cases of GCTs. Therefore, we suggest that serum VEGF levels have clinical significance as a biomarker of residual tumors in GCTs.

Previous studies have reported the efficacy of bevacizumab in GCTs [2, 3, 13, 16, 17]. In addition, it may be useful for controlling neovascularization-related symptoms, such as VEGF-mediated ascites in ovarian cancer [2], as shown in Case 2 in our study. The overall response and clinical benefit rates of bevacizumab in recurrent GCTs are 38% and 63%, respectively [3]. Thus, bevacizumab may be clinically important for controlling disease progression. However, as the insurance coverage for chemotherapy of GCTs is limited, our findings may be helpful for the selection of appropriate chemotherapy agents for aggressive GCTs.

In conclusion, serum VEGF levels may be of clinical importance as a biomarker for GCTs, and bevacizumab may be useful for recurrent GCTs. Further studies are required to evaluate the efficacy of serum VEGF levels and bevacizumab in patients with GCTs.

## Data Availability

The dataset supporting the conclusions of this article is owned by Teikyo University hospital but could be made available on request. Personal information will not be provided to ensure anonymity of the patient.

## Abbreviations

GCTs: Granulosa cell tumors
VEGF: Vascular endothelial growth factor
CT: Computed tomography
MRI: Magnetic resonance imaging
